# Executive functions, Personality traits and ADHD symptoms in adolescents: A mediation analysis

**DOI:** 10.1371/journal.pone.0232470

**Published:** 2020-05-06

**Authors:** Virginia Krieger, Juan Antonio Amador-Campos, Joan Guàrdia-Olmos

**Affiliations:** 1 Department of Clinical Psychology and Psychobiology, Faculty of Psychology, University of Barcelona, Barcelona, Spain; 2 Institute of Neuroscience, University of Barcelona (UBneuro), Barcelona, Spain; 3 Department of Social Psychology and Quantitative Psychology, Faculty of Psychology, University of Barcelona, Barcelona, Spain; 4 UB institute of Complex Systems, University of Barcelona, Barcelona, Spain; Universtiyt of Oviedo (Spain), SPAIN

## Abstract

Certain personality traits and cognitive domains of executive functions (EF) are differentially related to attention deficit hyperactivity disorder (ADHD) symptoms in adolescents. This study aimed to analyze the five-factor model (FFM) personality characteristics in adolescents with ADHD, and to examine whether EF mediate the relationships between FFM personality traits and ADHD symptoms. A comprehensive diagnostic assessment, including ADHD clinical interviews, ADHD rating scales, neuropsychological EF testing (i.e., working memory, flexibility and inhibition) and a personality assessment was carried out in a sample of 118 adolescents (75 ADHD and 43 control participants, 68% males), aged 12 to 16 years, and their parents and teachers. Adolescents with ADHD had lower scores than control participants on Conscientiousness and Agreeableness, and higher scores on Neuroticism. Structural equation models (SEM) showed that Conscientiousness directly influenced inattentive and hyperactive-impulsive symptoms, while Neuroticism, Agreeableness, and Extraversion directly affected hyperactive-impulsive symptoms. Only Conscientiousness exerted indirect effects on inattention, but not on hyperactivity-impulsivity symptoms, via EF; higher scores on Conscientiousness were related to higher scores on EF, which in turn were related to lower scores on inattentive symptoms. These findings corroborate the relationships between ADHD symptoms, FFM personality traits and EF and indicate the mediating effect of EF on the relationship between Conscientiousness and inattention.

## Introduction

Attention deficit hyperactivity disorder (ADHD) is a childhood onset disorder characterized by a persistent pattern of inattentive and hyperactive/impulsive behaviors, leading to three presentations: inattentive (ADHD-IA), hyperactive/impulsive (ADHD-HI), and combined (ADHD-C) [1].

ADHD symptoms reflect multiple causal pathways [2]. In particular, individual differences in executive functions (EF), temperament and personality traits may have a causal link to the emergence, variation, or persistence of ADHD symptoms. There is a solid body of evidence showing a clear involvement of executive processes in the core symptoms of ADHD. EF have been defined as “a family of cognitive control processes that operate on lower-level processes to regulate and shape behavior” [3 p. 893]. EF include cognitive processes such as working memory, inhibitory control and flexibility [4,5,6] and can be considered key contributors to the self-regulation of emotion, behavior and cognitive resources in top-down processes [7]. Several lines of research suggest that executive control requires flexible top-down or controlled information processing from the prefrontal executive areas [8,9]. Moreover, EF depend on the modulation of attention and emotion across bottom-up or automatic non-executive processes from limbic subcortical regions (e.g., amygdala) [7]. Thus, in a broader executive framework, top-down processes modulate, suppress or bias bottom-up information via EF [7,10]. There is also some evidence of developmental improvement in top-down and bottom-up connectivity associated with the increasing specialization of the prefrontal cortex (PFC) and limbic structures with age [11,12].

In children and adolescents with ADHD, several studies have shown links between top-down and bottom-up processes and ADHD symptoms [13,14,15]. In addition, it is possible that both top-down and bottom-up processes correlate differently with symptoms of both inattentive and hyperactive-impulsive types. Indeed, several studies have provided evidence for an association between top-down modulation and inattentive symptoms as well as an association between bottom-up modulation and hyperactive-impulsive symptoms in children and adolescents with ADHD [15,16,17]. Martel et al. [15] also reported relationships between top-down modulation and both ADHD symptom domains (i.e., inattentive and hyperactive-impulsive symptoms) in adolescents. Furthermore, a close examination of the measures assessing top-down and bottom-up regulatory processes suggests that though many individuals with ADHD perform poorly on most tasks that tap top-down or executive control appraisal processes [14,18,19,20], others do not present scores on these tasks that indicate clinically significant impairment [21,22].

In ADHD groups, EF has been traditionally evaluated with highly standardized and structured tasks that assess some top-down modulation, but not the affective or motivational component [7,5]. Thus, a growing body of research has shown that children and adolescents with ADHD are more likely than their peers to experience a degree of impairment in at least some performance-based measures of core EF skills [19,23,24]. Compared to controls, ADHD groups perform worse in cognitive tasks involving visuospatial working memory [25], inhibition [26], and flexibility [27]. In spite of this, given the heterogeneous nature of the underlying pathophysiological mechanisms of ADHD, there is no agreement on whether EF impairments are the core deficit in ADHD [28, 29].

### Personality traits and ADHD symptoms

Personality traits are defined as the broad range of individual differences in emotion, cognition and behavior that show relative stability over time, have biological bases and are organized hierarchically [30,31,32]. Currently, it is widely accepted that trait taxonomies such as the five-factor model (FFM) [33] provide a comprehensive account of the core characteristics of a personality structure [31,34], including characteristic adaptations or stable patterns of psychological functioning (e.g., attitudes, habits and skills), as well as self-concept [35]. These patterns and their configurations, for instance self-regulation skills, can be linked to specific dispositional personality traits [35]. According to the FFM, each of the five factors included in this model offers significant information on both normal and abnormal personality traits [35]. There is empirical evidence showing that the FFM provides a valid framework to understand specific links between personality traits and developmental psychopathology, including ADHD, in children and adolescents [36,37,38]. In addition, some main temperament traits show clear conceptual similarities with most FFM traits observed in children and adolescents [39,40].

The FFM groups personality traits into five broad domains: Conscientiousness, Neuroticism, Agreeableness, Extraversion and Openness, which are defined by more specific and narrow facets [31,33]. Conscientiousness is the most closely linked to planful behavior or executive control processes, while Neuroticism, Agreeableness and Extraversion are more closely linked to more reactive or motivational control processes [15,17].

Conscientiousness involves constraining impulses, effortful attention, planful behavior, organization and achievement goal orientation. The overarching inhibitory component of Conscientiousness is associated with self-regulation, probably reflecting some kind of top-down regulation process [41,42,43]. There is a link between low Conscientiousness and ADHD symptoms in some children and adolescents [15,44,45]. Nigg et al. [46], using personality data and examining adults, found that the inattentive symptoms of ADHD were related to low Conscientiousness. Additionally, the few studies carried out in this regard are quite consistent in suggesting that low Conscientiousness is associated with inattention in children [15,16] and hyperactivity-impulsivity in adolescents [15].

Neuroticism reflects the tendency towards excessive negative affectivity, emotional reactivity and distress, as well as low self-esteem. Agreeableness reflects compassion, empathy and cooperation whereas Extraversion reflects positive affect, assertiveness, activity level and sociability. Research to date suggests that some aspects of Neuroticism, Agreeableness and Extraversion (e.g., processing of emotional states or reward responses) reflect bottom-up regulation processes [15,17,40]. Many children and adolescents with ADHD present high levels of Neuroticism/Negative Emotionality and lower levels of Agreeableness compared to control subjects [38,44,45,47]. Accordingly, Martel et al. [15] reported that low Neuroticism and high Agreeableness were associated with fewer hyperactive-impulsive symptoms in children and adolescents with ADHD. Nigg et al. [46] found that high levels of Neuroticism correlated with inattentive symptoms, while hyperactive-impulsive symptoms were linked to lower levels of Agreeableness in adults. Moreover, low Agreeableness and high Neuroticism have been linked to the persistence of ADHD symptoms during adolescence [48]. The findings on the links between Extraversion and ADHD symptoms are mixed and somewhat less consistent across the literature. While some studies have shown no reliable association between ADHD symptoms and Extraversion in children, adolescents and adults [16,45,46], others have indicated significant links between hyperactive-impulsive symptoms and Extraversion [37,38,47]. Furthermore, in the meta-analysis of Gomez and Corr [37], positive emotionality (comparable to FFM/Extraversion) correlated with inattention, but not with hyperactivity-impulsivity.

In contrast to the four other personality traits, Openness, which refers to creativity, flexibility and willingness to explore new ideas and experiences, does not appear to be clearly linked to ADHD symptoms [37,48]. Based on the current evidence, no conclusive relationships can be established between Openness and ADHD symptoms in children and adolescents [48,49]. Furthermore, Conscientiousness, Neuroticism, Agreeableness and Extraversion, compared with Openness, have clearer conceptual links with dispositional temperamental traits and are more widely validated factors in models of child temperament and personality [15,39,50]. For their part, Conscientiousness and Openness are considered to be traits associated with self-regulatory abilities and appear to be related during childhood and adolescence, being less clearly dissociable than in adults [39,51].

### Personality traits, EF and ADHD symptoms

The research carried out to date has provided evidence supporting the hypothesized links between FFM personality traits, EF measures and ADHD symptoms [15,16]. Martel et al. [16] found a positive correlation between cognitive control measures (i.e., inhibition and response variability), Conscientiousness and inattention in children with ADHD. Furthermore, in a study of young children with ADHD, Martel [52] found that low Conscientiousness/Effortful control (EC) and high Extraversion/Surgency were related to impairments in neuropsychological EF measures (i.e., memory working, inhibition, and flexibility) and hyperactive-impulsive symptoms rated by parents and teachers. Another study also showed links between Conscientiousness/EC, some EF (i.e., inhibition and flexibility) and the inattentive and hyperactive-impulsive symptoms of ADHD in adolescents [15]. Conscientiousness and EC are related theoretically, since both reflect self-direct control [39], which seems to be grounded in executive functioning or top-down regulation [15,39,53]. In line with this idea, EC in adolescents was associated with cognitive EF and ADHD symptoms in a mediation analysis framework [54]. Indeed, a mediational effect of EC on the relationships between EF and ADHD symptoms in adolescents was observed by Krieger et al. [54], who found significant indirect effects of EF (i.e., working memory, planning and inhibition) via EC on the severity of both inattentive and hyperactive-impulsive symptoms.

Taken together, these findings suggest that EF, FFM personality traits and ADHD symptoms are interconnected in multiple ways. In fact, inattention (e.g., poor attention, deficits in planful behavior, and lower task persistence) is associated with executive control and Conscientiousness, reflecting underlying top-down regulation processes, while hyperactivity-impulsivity (e.g., overactivity, poor achievement goal orientation, impulsivity and higher emotional distress) is linked to Agreeableness, Neuroticism and reactive control, reflecting underlying bottom-up regulation processes [17].

Thus, these different patterns of associations between EF, FFM traits and ADHD symptoms may have two different causal pathways that mediate either more top-down executive influences or more bottom-up automatic influences. Consequently, individual differences in EF may reflect prefrontal top-down controlled influences, thus explaining the links between the FFM traits and ADHD symptoms in situations that demand more controlled than automatic responses. It is also crucial to consider that during adolescence, the neural systems (e.g., prefrontal and the anterior cingulate cortex) mediating top-down processes have not yet reached the level of that observed in adults [9]. The increase in top-down connectivity during this period enables improvements in the processing of more complex information and in the executive control of behaviors [9].

The current study aimed to extend previous research on the relationship between EF, dispositional traits (i.e., personality and temperament) and ADHD [15,16,54] during adolescence in a mediation framework, by assessing whether the potential pathways linking specific dispositional personality traits (i.e., FFM) to inattentive and hyperactive-impulsive symptoms are assembled via EF. Though few studies have explored the relationships between these variables, we expected to find an association between Conscientiousness, Agreeableness, Extraversion and Neuroticism and ADHD symptoms. In addition, we expected EF to mediate the link between Conscientiousness and inattention, given that they share similar neural substrates in the prefrontal cortex (i.e., top-down executive modulation). Thus, we hypothesized that: (1) the ADHD group would score lower on Conscientiousness and Agreeableness and higher on Extraversion and Neuroticism than the control group; and (2) Conscientiousness would have indirect effects on inattention and hyperactivity-impulsivity via EF. We did not expect the same effects for Agreeableness, Neuroticism and Extraversion because these are linked more to bottom-up modulation.

## Method

### Participants

The present study is a sub-study of a project assessing the relationships between EF, temperament and personality in adolescents with ADHD and controls. The sample includes all adolescents who participated in the study (118 participants aged between 12 and 16 years). The ADHD group comprised 75 participants (48 ADHD-IA and 27 ADHD-C, age: *M* = 13.60, *SD* = 1.31, 68% males), while the control group consisted of 43 participants (age: *M* = 13.42, *SD* = 1.38, 55.8% males). Using the recommendations of McNeish [55] in relation to sample size, we carried out a Bayesian estimation of sample error for each group: .03 [95% CI = 0.01–0.05] for ADHD groups and .05 [95% CI = 0.03–0.08] for the control group. Obviously, it was slightly larger in the control than the ADHD group.

Details of sample recruitment, clinical diagnosis assignment, comorbid disorders, and inclusion and exclusion criteria were described in detail elsewhere [54]. Almost all the adolescents (90.5%) lived in two-parent families. The parents’ educational level was distributed as follows: high school diploma (ADHD: 34.7%; controls: 25.6%), four years of college (ADHD: 26.4%; controls: 38.4%), education beyond college in professional training (ADHD: 21%; controls: 11.6%), and junior high and primary school (ADHD: 17.9%; controls: 24.4%). The ADHD and control groups did not differ significantly in terms of parents’ educational level, *χ*^2^ (71, *N* = 118) = 85.09, *p* = .12.

Final diagnostic assignment was determined by data from the Clinical Interview-Parent Report Form and T scores on the Conners-3 parent and teacher rating scales. Following the recommendations of Pliszka [56] for the assessment of ADHD, all cases considered to be potentially eligible were reviewed by a principal investigator and a panel of three ADHD experts (i.e., two clinical psychologists and one psychiatrist). Unanimous agreement of the panel was required for the assignment of the participants to the ADHD or control groups, and for comorbid diagnosis for all the participants (*N* = 118).

The ADHD group comprised all the individuals who had been assessed at two child and adolescent mental health centers and one university psychology service and who had been diagnosed with ADHD. Of 75 participants diagnosed with ADHD, 24 (32%) had no other comorbid diagnosis; 11 (14.7%) had one comorbid disorder (10 internalizing disorder and one externalizing disorder), and 40 (53.3%) had two or more comorbid disorder (10 internalizing disorders and 30 internalizing and externalizing disorders). Ten participants with ADHD (7.5%) who were being treated with stimulant medication for ADHD symptoms were asked to discontinue their medication for a period of 24 hours before each testing session, under the supervision of the treating physician and their parents. No other participants in the ADHD or control group were taking other psychotropic medication.

In order to recruit participants for the control group, meetings were held with the parents and teachers of the secondary school, informing them of the objectives of the study and inviting them to participate.

### Ethical standards

This study was reviewed and approved by the Medical Research Ethics Committee at the Centre de Salut Mental de Les Corts (Barcelona) and by the Research Ethics Committee of the University Psychology Service (University of Barcelona) and the Salesians School in Badalona (Barcelona). Participation was voluntary in all cases and no financial compensation was offered. Parents, teachers and participants were informed of the aims, duration and procedure of the study. Verbal informed consent was obtained from participants and written informed consent was obtained from their parents or legal guardians before study entry. The study complied with the principles of the 1975 Declaration of Helsinki (revised in Tokyo in 2014).

### Instruments

#### Assessment of ADHD symptoms

Clinical Interview-Parent Report Form [57]. This instrument records information from parents of children and adolescents about parental concerns, reasons for the assessment, family environment and history (psychopathological background of parents and siblings); health, temperament, school and social history of the child evaluated, and review of DSM disorders (ADHD, defiant oppositional/negative, dissocial, bipolar, anxiety and mood). ADHD symptoms were assessed on a Likert scale ranging from 0 (never, seldom) to 3 (often, very frequently). The symptom was considered absent if it was scored 0 or 1 and present if it was scored 2 or 3. Reliability of symptom counts was high in this sample (inattention, α = .94; hyperactivity-impulsivity, α = .93)

Conners scales, 3rd Edition (Conners 3) [58]. This rating scale assesses ADHD behaviors, learning problems, defiance-aggression and related problems, and comorbid conditions in children and adolescents. The two long forms were used: the parent (Conners 3-P: 6–18), and teacher versions (Conners 3-T; 6–18. In our sample, in agreement with previous analyses [54] the reliability (internal consistency) was good.

#### Performance-based measures of EF

The spatial memory subtest (SSp) of the Wechsler Nonverbal Scale of Ability (WNV) [59]. This test assesses spatial working memory through a task which involves reproducing a visuomotor sequence in the same order as that presented by the examiner (Span Forward; SSpF index) or in reverse order (Span Backward; SSpB index). Reliability (internal consistency) in this sample was good [54].

Rey-Osterrieth Complex Figure Test (ROCF) [60,61]: This test evaluates visual spatial/ constructional processing, and immediate and delayed visual memory through a task requiring the copying of a complex figure (ROCF-C) and its later reproduction from memory (RCFT-M). Reliability (Kendall coefficients) was excellent in this sample [54].

Porteus Maze Test (PMT) [62]. This test assesses the ability to anticipate, plan, and inhibit behaviors. The task is to find the way through a series of 12 mazes of increasing difficulty, without lifting the pen or entering a dead end. Reliability (internal consistency) in this sample was acceptable [54].

Wisconsin Card Sorting Test (WCST) [63]. The test assesses reasoning, concept formation, and the ability to change problem-solving strategies (flexibility). Reliability (internal consistency) in this sample was acceptable [54].

Trail Making Test [64]. The test assesses visual scanning, attention, and cognitive flexibility. It comprises two tasks: task A requires the subject to join up 25 circles, distributed over a sheet of paper, and numbered consecutively, in ascending order as quickly as possible: in task B the subject joins up numbers and letters, alternately, in ascending order (e.g., 1-A; A-2 until L-13). Reliability (internal consistency) in this sample was acceptable [54].

The d2 Test of Attention [65]. This test consists of a set of letters, p or d, which have some small dashes arranged individually or in pairs either above or below each letter. The task is to cross out only the d with two dashes, regardless of whether the dashes appear above or below the letter or one above and one below. Commission errors and total test effectiveness are taken as indicators of inhibition. Reliability (internal consistency) in this sample varies between acceptable (commission errors) and good (total test effectiveness) [54].

EF measures were grouped according to the hypothetical common underlying cognitive process [66,67,68]. Table 1 shows a summary of performance-based EF measures, score type, factor loadings and percentage of variance explained by each cognitive domain.

**Table 1:** Summary of performance-based EF measures.

EF	Cognitive EF task	Measure and score type	Factor loadings PCA	Total explained variance
Working Memory	Spatial span (SSp) of Wechsler Nonverbal scale of ability (WNV)	SSp Forward, raw scores	.72	48.09%
		SSp Backward, raw scores	.76	
	Rey-Osterrieth Complex Figure Test (ROCF)	Immediate recall accuracy, percentile scores	.60	
Flexibility	Wisconsin Card Sorting Test (WCST)	Perseverative errors, standardized T scores	.85	59.03%
		Conceptual level responses, standardized T scores	.94	
		Number of categories completed, standardized T scores	.83	
	Trail Making Test (TMT)	Total time in seconds’ part B, raw scores	-.26	
Inhibition	PMT	Qualitative *Q* score	.76	55.0%
	d2 Test of Attention	Commission errors, percentile scores	.77	
		Total test effectiveness, percentile scores	-.72	

EF: Executive functions; PCA: Principal Component Analysis. Neuropsychological measures were grouped according to the hypothetical underlying cognitive process that they engage [66, 67,68].

### Personality

Big Five Questionnaire for Children (BFQ-C) [69]. This self-report assesses Conscientiousness, Openness/Intellect, Extraversion/Energy, Agreeableness, and Neuroticism/Emotional Instability in children and adolescents. The BFQ-C consists of 65 items (13 for each personality factor). The reliability coefficients of the Spanish adaptation (BFQ-NA) [70] ranged from α = .78 (Emotional Instability) to α = .88 (Conscientiousness). The Conscientiousness, Agreeableness, Neuroticism and Extraversion T-scores were considered here. Openness was not taken into account in subsequent analyses because this trait does not present a clear conceptual link with dispositional temperamental traits [39] or any conclusive relationships with ADHD symptoms [48].

### Procedure

After agreeing to participate and signing the informed consent, a clinical interview was administered to the parents or legal guardians of all participants in the first stage for stablishing the clinical diagnosis. The assessment protocol also includes measures of intelligence, behavioral executive functions and temperament. These data are described elsewhere and are not part of the current analyses. A more detailed and extensive description of multi-step assessment processes can be found elsewhere [23, 54]. All participants completed the evaluation procedure. Once the assessment process was complete, all participants received a written report with their individual results. In addition, participants in the ADHD group attended a feedback session in the presence of their parents and the reference health professional.

### Data analysis

Differences between groups according to gender and age were examined using the chi-square test and the t-test respectively. Since all three cognitive EF domains [working memory (WM), flexibility (FL), and inhibition (IN); Table 1] grouped tasks with different score types (i.e., percentile, standard or raw scores), the scores were transformed into Z-scores. To obtain a single score for each cognitive EF domain a principal component analysis (PCA) was performed for each of these domains, forcing the solution to extract one component. To avoid the risk that other factor models might fit better to the one-dimensional model, possible Seed-ESEM models were analyzed in a complementary way to rule out better factor solutions, but none were found. The factor scores for each cognitive EF factor (i.e., WM, FL, and IN) were obtained using Bartlett's method of regression, and they were thus considered as weighted Z-scores (Table 1). T-scores of BFQ-C and Conners 3 (inattentive and hyperactive-impulsive symptoms) parent reports were transformed into Z-scores. For all the analyses, Z-scores of EF domains, FFM personality traits and ADHD symptoms of total sample were used. Differences according to age and gender in FFM personality traits were analyzed by a factorial ANOVA. Separate MANCOVAs were carried out to analyze differences between the groups in FFM personality traits, controlling for comorbidities (i.e., internalizing: anxiety and depression, and externalizing: oppositional defiant disorder, and conduct disorder). Standardized mean difference (Cohen’s *d*) effect sizes were calculated based on average and standard deviations of each group in order to assess differences between groups on EF and the FFM personality traits. The Cohen’s *d* [71] guidelines for interpreting effect size criteria were also used: 0.20, small; 0.50, medium, 0.80, large. Cohen’s *d* values of 1.30 were considered very large [72]. Pearson correlation coefficients were calculated to explore the relationships between the variables, taking into account the following effect size criteria: *r* = .10 to .29, low; *r* = .30 to .49, moderate; *r* = .50 to 1.0, high [73]. Finally, Cohen’s [73] effect size criteria for eta squared (η^2^) were also used: 0.01–0.05, small; 0.06 - .13, medium, and ≥ .14 large.

In this study, the total sample was considered because we believe that EF performance, FFM traits and both inattention and hyperactivity-impulsivity behaviors exist along a wide spectrum in which different subpopulations range from extreme (abnormal) to normal behaviors, reflecting more quantitative rather than qualitative differences [45,74,75]. In addition, the entire sample was used because we did not want to consider the ADHD variable dichotomously, since the measures used are interval scales; therefore, it is assumed that there is no absolute value. Consequently, we considered it much more interesting to use the values of the distribution observed in its original form, not dichotomously identifying controls and cases. It is important to indicate that the control subjects present scores other than 0, so we propose to identify ADHD as a continuum derived from the psychometric properties of the scale used. Hence, the impact of mediation was estimated from the Structural Equation Modeling (SEM) structural coefficients using the variable as a discrete quantitative distribution. The total sample was submitted to SEM analysis in order to explore the variability of the outcomes across core symptoms of inattention and hyperactivity-impulsivity with respect to FFM traits and EF. In line with this idea, some studies have suggested that SEM assumes probabilistic causality more than deterministic causality [76,77]. Consequently, we expected the SEM to help us explore the associations between EF, the FFM personality traits and ADHD symptoms (i.e., inattentive and hyperactive-impulsive).

The data analysis strategy for testing the path models was guided by the recommendations of Hayes [78], with Mplus 7.3 for Mac OS being used to test the hypothesis. We composed a latent variable of EF using WM, FL and IN. To estimate the direct and indirect total effects of the hypothetical relationship between EF, Conscientiousness, Neuroticism, Agreeableness and Extraversion on inattentive and hyperactive-impulsive symptoms, a set of path analyses was fitted using SEM. Thus, only one model was tested, considering the latent variable of EF (i.e., WM, FL and IN) as a mediator in the path among the FFM personality traits (i.e., Conscientiousness, Neuroticism, Agreeableness and Extraversion) and inattentive and hyperactive-impulsive symptoms. In this model, a change in EF was hypothesized to mediate the indirect effects of Conscientiousness on inattention, but not the indirect effects of Neuroticism, Agreeableness and Extraversion on inattention and hyperactivity-impulsivity. We also considered a path to estimate the supposed link between the two symptom dimensions (i.e., inattentive and hyperactive-impulsive).

The robust maximum likelihood estimation in Mplus 7.3 [79] was used to estimate the model parameters since this method can be used to fit non-normally distributed data [76]. Since χ^2^/df is sensitive to sample size [80], we also assessed model fit by calculating the following fit indices: the standardized root mean squared residual (SRMR) and the root mean square error of approximation (RMSEA), the Tucker-Lewis Index (TLI), and the comparative fit index (CFI). Thus, we considered the following indices for acceptable fit [χ^2^/df between 2 and 5; RMSEA (or SRMR) ≤ .08; CFI ≥ .90 and TLI > .90] and for good fit [χ^2^/df < 2; RMSEA (or SRMR) ≤ .06; CFI ≥ .95 and TLI > .95]. According to the observed distribution of ADHD using the whole distribution (not for each group), indirect effects and standard errors were estimated with the bootstrapping method (bootstrap replicates, 10.000) to obtain more accurate confidence intervals and, in this way, reduce the effects of the violation of the multivariate normal distribution of the observed variables involved in the model [81,82]. Regarding the sample size, some studies [83,84] suggest that an acceptable sample size depends on the structure and nature of the data: the stronger the data, the smaller the sample size required to reproduce them in a model.

In fact, as noted by Wolf et al. [84], to uncover mediation or indirect effects in some cases, complex models with strong data may require smaller samples sizes. As is already known, it is easy to obtain the number of parameters to be estimated from the degrees of freedom, the sample size being sufficient for each parameter and the standardized estimates having small standard errors. In our case, we complied with the rule of Wolf et al. [84] in terms of the sample to parameter ratio (4:1), while also having small estimates of the standard errors (ranging from 0.06 to 0.14). Thus, we considered that our sample size was sufficient for robust estimates.

## Results

Full details of sample characteristics, measures of ADHD symptoms and cognitive EF can be found elsewhere [54], but the following points are relevant to this study. ADHD and control groups did not show differences in age or gender. There were significant differences between the ADHD and control groups in ADHD symptoms with very large effect sizes for inattention (*d* = 3.75) and hyperactivity-impulsivity (*d* = 2.90). There were no significant age and gender differences for cognitive EF. There were also no significant age and gender differences for the FFM personality traits [age (Conscientiousness: *F*(4, 113) = 1.43, *p* = .21; Agreeableness: *F*(4, 113) = 1.13, *p* = .34; and Extraversion: *F*(4, 113) = 0.27, *p* = .89) and gender (Conscientiousness: *F*(1, 116) = 3.76, *p* = .06; Neuroticism: *F*(1, 116) = 2.91, *p* = .09; and Extraversion: *F*(4, 113) = 0.27, *p* = .89)]. However, there were significant age differences for Neuroticism (*F*(4, 113) = 4.06, *p* = .001). A factorial ANOVA revealed that the group effect for age remained statistically significant (*F*(2,112) = 5.63, *p* = .005), with 14- and 15–year-old adolescents scoring significantly higher (*M* = 54.98, *SD* = 1.58) than 12- and 13–year-old adolescents (*M* = 47.89, *SD* = 1.45). The interaction effect between age and gender was not significant (*F*(2,112) = 0.21, *p* = .80). Additionally, there were significant gender differences for Extraversion (*F*(1, 116) = 7.76, *p* = .01) and Agreeableness (*F*(1, 116) = 8.17, *p* = .01). A factorial ANOVA showed that the group effect for gender did not remain statistically significant for Extraversion (*F*(1, 112) = 2.78, *p* = .09) and Agreeableness (*F*(1, 112) = 2.19, *p* = .14). The interaction effect between age and gender was not significant for both Extraversion (*F*(2,112) = 1.34, *p* = .26) and Agreeableness (*F*(1, 112) = 1.21, *p* = .30).

### Performance-based EF measures

Performance for each executive domain (i.e., WM, FL and IN) for both the ADHD and control participants were documented in great detail elsewhere [54]. Adolescents with ADHD presented significantly lower scores than controls in WM and IN, with medium effect sizes for WM (*d* = 0.78) and large effect sizes for IN (*d* = 1.24). In contrast, there were no significant differences between the ADHD and control groups for FL.

### Personality traits

Descriptive statistics, Cohen’s *d* effect size and MANCOVA analysis for the two groups in personality traits are shown in Table 2.

**Table 2:** Means, standard deviation, Cohen’s d effect size and MANCOVA analysis of ADHD and control groups on BFQ-C self-report measures.

	ADHD (n = 75)	CG (n = 43)			Univariate	
BFQ-C personality factors	Mean (SD)	Mean (SD)	*t*	*df*	*p*	*Cohen’s d*
Conscientiousness	-.474 (.684)	.827 (.925)	8.047	116	.001	1.668
Extraversion	-.052 (.981)	.091 (1.03)	0.753	116	.453	0.139
Agreeableness	-.252 (.893)	.439 (1.03)	3.816	116	.001	0.731
Neuroticism	.172 (.972)	-.301 (.987)	2.533	116	.01	0.470

ADHD: attention deficit hyperactivity disorder; CG: control group; SD: (Standard deviation); *d*: Cohen’s *d* effect size; *df*: degree of freedom.

Adolescents with ADHD showed significantly lower scores than control subjects on Conscientiousness and Agreeableness, and higher scores on Neuroticism (Table 2). The effect sizes were small for Neuroticism (*d* = 0.47), medium for Agreeableness (*d* = 0.73) and very large for Conscientiousness (*d* = 1.66). MANCOVAs were performed to compare the FFM personality traits between the ADHD and control groups, with comorbidities included as covariates. MANCOVAs of Conscientiousness, Agreeableness and Neuroticism using Pillai’s trace revealed that after controlling for comorbidities, the group effects remained significant: V = .37, *F*(3, 113) = 21.99, *p* = .001, η^2^ = .37. Thus, the effect of comorbidities was not significant. There were no differences between the ADHD and control groups for Extraversion.

### Relationships between personality traits, cognitive EF factors, and ADHD symptoms

Table 3 shows Pearson correlations between FFM traits (i.e., Conscientiousness, Neuroticism, Agreeableness and Extraversion), cognitive EF, and inattentive and hyperactive-impulsive symptoms for the ADHD and control groups. Conscientiousness had a low-to-moderate positive correlation with IN (*r*(117) = .33, *p* < .001) and a high-to-moderate negative correlation with inattention (*r*(117) = —.60, *p* < .001) and hyperactivity-impulsivity (*r*(117) = —.37, *p* < .001). Neuroticism had a low negative correlation with WM (*r*(117) = —.21, *p* < .05) and IN (*r*(117) = —.19, *p* < .05), and a moderate positive correlation with inattention (*r*(117) = .30, *p* < .001) and hyperactivity-impulsivity (*r*(117) = .31, *p* < .001). Finally, Agreeableness only showed low and negative correlations with inattentive symptoms, *r*(117) = —.28, *p* < .001 and hyperactive-impulsive symptoms, *r*(117) = —.21, *p* < .001. No significant correlations were found between Extraversion and Agreeableness and EF. Inattentive and hyperactive-impulsive symptoms showed negative and low-to-moderate correlations with EF, ranging from *r*(117) = —.23, *p* < .01 (IN and hyperactive-impulsive symptoms) to *r*(117) = —.43, *p* < .001 (IN and inattentive symptoms).

**Table 3:** Pearson correlations between cognitive EF factors, personality factors BFQ-C self-report and inattentive and hyperactive-impulsive symptoms of Conners-3 parent report (DSM subscales) for ADHD and control groups (*N* = 118).

	1	2	3	4	5	6	7	8	9
1. Working memory	1								
2. Flexibility	.11	1							
3. Inhibition	.57**	.18	1						
4. Conscientiousness	.15	.08	.33**	1					
5. Extraversion	-.11	.02	-.09	.25**	1				
6. Agreeableness	-.01	.07	.07	.57**	.53**	1			
7. Neuroticism	-.21^*^	.04	-.19^*^	-.26**	.10	-,07	1		
8. Inattentive	-.31**	-.12	-.43**	-.60**	-.10	-.28**	.30**	1	
9. Hyperactive-impulsive	-,14	-,05	-,23^*^	-,37**	,03	-,21^*^	,31**	,56	1

ADHD: attention deficit hyperactivity disorder; Inattentive: Conners-3 DSM ADHD inattentive Scale; Hyperactive-impulsive: Conners-3 DSM ADHD hyperactive-impulsive Scale. In bold type, significant correlations between cognitive EF, personality factors, and ADHD symptoms.

* *p* < .05

** *p* < .01.

### Assessing the mediating role of EF between FFM personality traits and inattentive and hyperactive-impulsive symptoms

To analyze the role of EF as a possible mediator between the FFM traits and ADHD symptoms, a mediational model was constructed. We examined only one model containing four mediation pathways, with Conscientiousness, Neuroticism, Agreeableness and Extraversion indirectly related to inattention and hyperactivity-impulsivity via cognitive EF. We also examined three pathways with Neuroticism, Agreeableness and Extraversion directly related to hyperactivity-impulsivity. Here, we allowed inattentive-hyperactive-impulsive symptoms to be correlated. Thus, following the usual criteria for SEM, the model that included Conscientiousness, Neuroticism, Agreeableness and Extraversion had a good global fit: χ^2^ = 16.89, *df* = 12, *p* > .15; CFI = 0.98; TLI = 0.96; RMSEA = 0.05 (90% CI = 0.00–0.11) and SRMR = 0.03 (see Table 4). The analysis of factor loadings was also performed for the EF, and each path was statistically significant: WM (λ = .75, SE = .06, p ≤ .001), FL (λ = .69, SE = .06, p ≤ .001) and IN (λ = .75, SE = .08, p ≤ .001). In addition, the EF model explained 56% (p ≤ .001) of the variance for WM, 57% (p ≤ .001) of the variance for IN and 48% (p ≤ .001) of the variance for FL.

**Table 4:** Goodness-of-fit indices for personality traits (*N* = 118).

Model	χ^2^/*df*	CFI	TLI	RMSEA (90% CI)	SRMR
C, E, A, N	1.40	0.98	0.96	0.05 (0.00–0.11)	0.03

χ2: Chi square index fit; df: degrees of freedom; CFI: comparative fit index; TLI: Tucker-Lewis Index; RMSEA: root mean square error of approximation; 90% CI: 90 confidence intervals; SRMR: standardized root mean squared residual; C: Conscientiousness; E: Extraversion; A: Agreeableness; N: Neuroticism.

**Fig 1:**
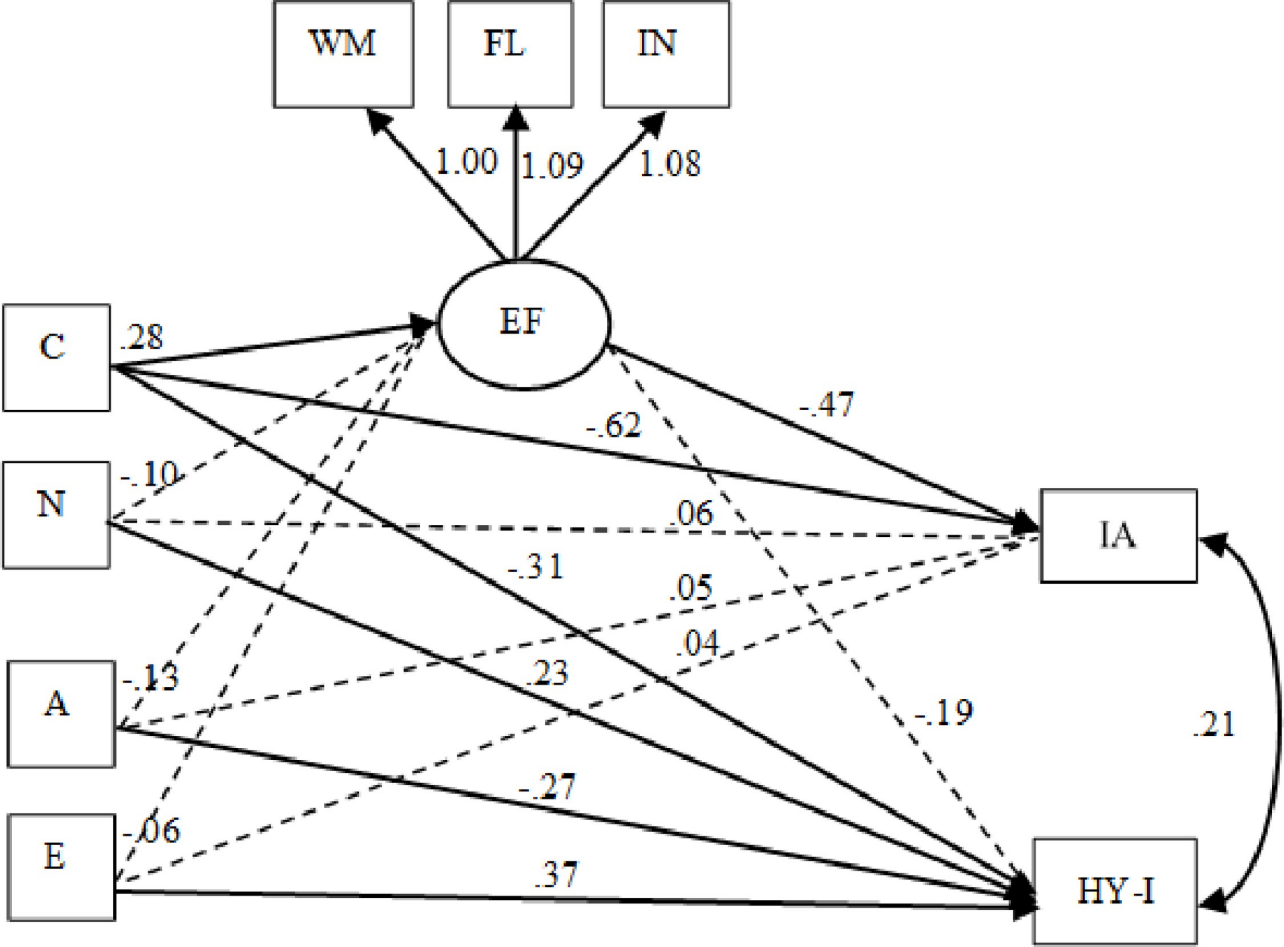
Structural equation model of relationship between cognitive executive function, personality traits and inattentive and hyperactive-impulsive symptoms. N = 118. Solid lines show unstandardized paths coefficients, significant at the *p* < .001 level. Dashed lines are non-significant paths. EF: executive function; WM: working memory; FL: flexibility; IN: inhibition. Personality traits; C: Conscientiousness; N: Neuroticism; A: Agreeableness; E: Extraversion. IA: inattentive, HY-I: hyperactive-impulsive.

The results of the mediational models revealed that Conscientiousness (ß = -0.61; *SE* = 0.09, 95% CI [-0.85, -0.35], *p* = .001), but not Neuroticism (ß = 0.06; *SE* = 0.07, 95% CI [-0.12, 0.25], *p* = .37), Agreeableness (ß = 0.05; *SE* = 0.08, 95% CI [-0.17, 0.28], *p* = .52) or Extraversion (ß = 0.04; *SE* = 0.07, 95% CI [-0.13, 0.22], *p* = .55), had significant direct effects on inattention. The direct effects of Conscientiousness (ß = -0.30; *SE* = 0.11, 95% CI [-0.57, -0.02], *p* = .005), Neuroticism (ß = 0.23; *SE* = 0.08, 95% CI [0.00, 0.44], *p* = .006), Agreeableness (ß = -0.27; *SE* = 0.10, 95% CI [-0.53, 0.00], *p* = .007) and Extraversion (ß = 0.37; *SE* = 0.08, 95% CI [0.15, 0.57], *p* = .001) on hyperactive-impulsive symptoms were significant. Additionally, one indirect pathway of Conscientiousness on inattention via EF was significant (ß = -0.13; *SE* = 0.06, 95% CI [-0.34, -0.01], *p* = .03). The indirect effects of Neuroticism (ß = 0.05; *SE* = 0.04, 95% CI [-0.05, 0.17], *p* = .23), Agreeableness (ß = 0.06; *SE* = 0.05, 95% CI [-0.05, 0.24], *p* = .21) and Extraversion (ß = 0.03; *SE* = 0.04, 95% CI [-0.07, 0.17], *p* = .44) on inattention through EF were not statistically significant. Furthermore, no significant indirect effects of Conscientiousness (ß = -0.05; *SE* = 0.05, 95% CI [-0.24, 0.04], *p* = .31), Neuroticism (ß = 0.02; *SE* = 0.02, 95% CI [-0.02, 0.12], *p* = .43), Agreeableness (ß = 0.02; *SE* = 0.03, 95% CI [-0.02, 0.18], *p* = .44) and Extraversion (ß = 0.01; *SE* = 0.02, 95% CI [-0.03, 0.14], *p* = .60) on hyperactivity-impulsivity through EF were found. This model explained up to 58% of the variance for inattentive symptoms (*R*^*2*^ = 0.58) and 41% of the variance for hyperactive-impulsive symptoms (*R*^2^ = 0.41). Thus, EF mediate the association between Conscientiousness and inattention in adolescents. The final mediational models are shown in Fig 1.

## Discussion

Individual differences in EF and personality traits are central components of self-regulation, which is increasingly recognized as a core aspect in many behavioral outcomes in adolescents with ADHD. The present study explored personality traits associated with ADHD symptoms in adolescents. In addition, we aimed to determine whether EF could explain the relationships between personality traits and ADHD symptoms. To this end, we tested the indirect effects of the personality traits (i.e., Conscientiousness, Neuroticism, Agreeableness and Extraversion) on inattention and hyperactivity-impulsivity via EF (i.e., working memory, flexibility and inhibition).

Our data partially confirmed our first hypothesis, namely that the ADHD group would score lower on Conscientiousness and Agreeableness and higher on Extraversion and Neuroticism. We observed an association between ADHD and lower scores on Conscientiousness and Agreeableness and higher scores on Neuroticism. There was no significant correlation between ADHD and Extraversion. These findings are consistent with those of previous studies examining personality traits in children and adolescents with ADHD and reporting low Conscientiousness and Agreeableness and high Neuroticism [15,16,35,37]. Furthermore, these findings agree partially with those of Gomez and Corr [37] and Tackett et al. [38] who reported significant relationships between ADHD and low scores on Conscientiousness and Agreeableness and high scores on Neuroticism and Extraversion in both children and adults. In our study, the fact that we had more cases of ADHD-IA in our ADHD group may support these results since inattentive symptoms appear to be more specifically related to Conscientiousness and, as reported in some studies, also to Neuroticism [38,46].

Importantly, we observed associations between ADHD and some of the personality traits closely related to self-regulation abilities that are considered impaired in this disorder [35,47,85]. For example, high impulsivity and disrupted goal-directed behavior may be related to lower Conscientiousness and Agreeableness, and higher Neuroticism, which would probably explain some of the breakdowns in the self-regulation abilities of children and adolescents with ADHD [35,38,86,87]. In this sense, some studies have pointed out that Conscientiousness, Neuroticism (reversed) and Agreeableness are more closely linked to self-regulation in cognitive, emotional and behavioral domains [30,35,88]. In support of this argument, our findings, analyzing the whole sample, showed significant and lower correlations between Conscientiousness (*r* = .33) and Neuroticism (*r* = -.19) and inhibition. Hoyle and Davisson [87] suggested that the shared variance between (low) Neuroticism, Agreeableness and Conscientiousness is due to all three being closely related to the stability factor that reflects self-control tendencies by inhibition. Furthermore, several studies have found that personality traits differently explain the ADHD symptom domains, with Conscientiousness accounting more for inattention and Neuroticism and Agreeableness accounting more for hyperactivity-impulsivity [15,47].

Importantly, Extraversion did not appear to differ between the ADHD and control groups, as other studies with children and adolescents have found [37,44]. This is interesting since Extraversion, together with Conscientiousness and Neuroticism, are closely related to impulsive behaviors [86]. Impulsivity is a key component of ADHD, especially of the combined ADHD presentation [89], while Extraversion appears to be more specifically associated with hyperactivity-impulsivity [38]. In our sample, the lower number of participants with ADHD-C could have influenced the results. Additionally, our findings differ from those of Martel et al. [49], who found that children and adolescents with ADHD scored higher on Extraversion than control subjects. This discrepancy is probably due to the fact that our ADHD sample was smaller, which could have prevented us from detecting subtle differences in Extraversion between the ADHD and control groups [49].

In summary, our findings suggest substantial links between lower Conscientiousness and Agreeableness and higher Neuroticism with ADHD symptoms. No differences were found between the ADHD and control groups in Extraversion, thus corroborating the suggestion of De Pauw and Mervielde [45] that some sociability and activity contents related to Extraversion facets are expressed differentially in ADHD children. This suggests that individual differences in developmental changes in the FFM personality traits (i.e., self-regulation) might be a risk factor or influence both the emergence of ADHD symptoms and the degree of ADHD persistence into adolescence (i.e., Neuroticism) [46,48].

With respect to our second hypothesis, namely that EF may mediate the relationship between Conscientiousness and inattention, our findings indicated the indirect effect of this personality trait on inattention via EF. Thus, as we had tentatively posited, mediation analysis revealed that EF explained the relationship between Conscientiousness and inattention in the whole sample of adolescents. Specifically, higher scores on Conscientiousness were related to higher scores on cognitive EF performances, which in turn were related to lower scores of inattentive symptoms. Similarly, Krieger et al. [54] found that EC, related to Conscientiousness [90], mediated the effects of EF (i.e., working memory, planning, flexibility and inhibition) on inattention and hyperactivity-impulsivity symptoms in adolescents. This finding is particularly relevant in view of the increasing evidence that cognitive EF are related to EC empirically [15,54], and that both are closely associated with top-down aspects of self-regulation [15] which also underlie personality traits (i.e., Conscientiousness) and inattentive symptoms [16,91]. These results are consistent with those of previous studies demonstrating links between a latent factor for top-down regulation involving both Conscientiousness and EF (i.e., inhibition and flexibility) and inattention in children and adolescents with ADHD [15,16,17]. Our findings also partly agree with those of Martel et al. [15], who found an association between a latent factor of top-down regulation (i.e., Conscientiousness and EF measures) and both inattention and hyperactivity-impulsivity in adolescents. However, there were also direct effects of Conscientiousness on hyperactive-impulsive symptoms. Thus, dimensional traits such as Conscientiousness/EC appear to be important in the expression of both inattentive and hyperactive-impulsive symptoms during adolescence.

Additionally, individual differences in EF, involving top-down control mechanisms, could be associated with a breakdown in this pathway, explaining the relationship between Conscientiousness and only inattention, since all of them tap into self-regulation processes. Thus, when top-down processes become less available, EF become less efficient and the self-regulation processes, driven by mechanisms underlying Conscientiousness [43], are less able to regulate inattention. This may be due to the fact that both Conscientiousness and executive control processes (i.e., working memory and inhibition) are closely related to PFC pathway activation, which provides top-down regulation of a wide range of executive and voluntary behavioral control [9,92,93]. Findings from fMRI studies suggest that there are dysfunctions in some regions of the PFC and the anterior cingulate cortex that are associated with these top-down processes in individuals with ADHD [94].

In line with our hypotheses, there were no mediation effects between Neuroticism, Agreeableness and Extraversion and inattention and hyperactivity-impulsivity. Here, the mediation effects of EF were not considered since these FFM personality traits are linked more to bottom-up modulation (non-executive processes), as has been reported previously [15,17]. In fact, we observed direct effects of Neuroticism, Agreeableness and Extraversion on hyperactivity-impulsivity. Therefore, these findings suggest that individual differences in these FFM traits that involve, for instance, emotional reactivity and reward expectancies arise from bottom-up pathways (i.e., the amygdala and posterior cortical regions), explaining their association with hyperactive-impulsive symptoms (e.g., overactivity and impulsivity) [15,95].

Consistent with our second hypothesis, our findings indicated that EF account significantly for the relationship between Conscientiousness and inattention and that, together with EF, they are the best predictors of inattention. Similar results were reported by Krieger et al. [54] who found that EF together with EC (related to Conscientiousness) are good predictors of the two ADHD symptom domains in adolescents. This suggests that neural brain networks of top-down processing probably serve as a common framework for Conscientiousness/EC, EF and inattention in the service of cognitive self-regulation. In addition, Neuroticism, Agreeableness and Extraversion are the best predictors of hyperactivity-impulsivity, suggesting common bottom-up regulation processes involved in emotional reactivity. In line with these findings, Nigg [17] argued that ADHD is a combination of difficulties in the top-down and bottom-up control of information processing. Therefore, these patterns of relationships between the FFM personality traits, EF, and inattentive and hyperactive-impulsive symptoms highlight the complexity and clinical heterogeneity of ADHD in adolescents.

Our results suggested that adolescents with ADHD showed personality characteristics that are related to self-regulation difficulties. The mediation analysis indicated that EF account for the association between Conscientiousness and inattention but not with hyperactivity-impulsivity. These findings are consistent with the notion that core dispositional personality traits (e.g., FFM) and executive control processes are related and work together to guide and self-regulate behaviors [96]. Moreover, these results provide partial support to a small number of studies that have considered a dual pathway (i.e., top-down and bottom-up control) to explain the psychological processes that probably underlie ADHD [15,16,17,49]. Taken together, our findings are in line with the literature suggesting that measures of dispositional traits such as personality (i.e., FFM traits) and temperament (i.e., EC), and of cognitive processes (i.e., EF), can broaden our understanding of the clinical heterogeneity of ADHD [17,54].

### Implications for clinical practice

From a clinical perspective, and given the heterogeneity of ADHD, it is difficult to assign clear profiles of executive performance and personality traits in ADHD adolescents. Thus, clinical assessment of adolescents with ADHD should combine the use of EF and personality measures, which would make it possible to clarify clinically relevant issues at the level of normative and atypical cognitive and affective development. This could shed light on the factors that increase the risk of comorbidity in ADHD (e.g., Neuroticism) and provide guidelines for the development of individually tailored treatments targeting executive proficiency and self-regulation skills. In addition, an understanding of the profiles of EF and personality traits in children and adolescents might lead to the identification of risks and protective factors that help to predict and improve long-term adult outcomes. Also, in terms of improving clinical communication, describing a patient in terms of personality traits may be clearer and more useful for clinical decision-making than describing him or her solely through diagnostic labels such as those used by diagnostic systems [1,97,98]. It is worth stressing that although the use of self-reports in adolescents remains controversial (due to possible problems such as acquiescence bias), adolescence represents a valuable period for assessments [42]. In this study, the self-reports of BFQ-C were useful for describing FFM personality characteristics in adolescents.

### Limitations of the study

This study did have certain limitations that restricted its generalizability. A key limitation is the use of a cross-sectional design, meaning that it is impossible to demonstrate causality for the direction of the associations; therefore, caution must be taken when interpreting the results. A longitudinal, prospective study with more stringent testing would provide firmer conclusions about mediation. Furthermore, due to the complex nature of the etiopathogenetic pathways underlying ADHD, the most that we can infer is that the analysis of the directional relationships tested in our mediational model is only one approach among many other different levels of analysis in the search for a better understanding of the developmental pathways associated with ADHD during adolescence. Thus, it may be important in future research and replications to assess the age-related changes in this model across the lifespan. We also chose to examine a single model with four personality traits (i.e., Conscientiousness, Extraversion, Agreeableness and Neuroticism) as simultaneous independent predictors; we excluded Openness from the model because it has not been well validated in models of child temperament and personality. Therefore, it is possible that we did not obtain comprehensive characteristics of the structure underlying the personality traits during adolescence or of the Openness and EF associations, which further limits the generalization of our results. Additionally, our sample size and the percentage of participants in each of the groups (63.6% with ADHD and 36.4% controls) restricted our ability to fully understand the nature of the intergroup differences. Lastly, the ADHD group did not include individuals with hyperactive-impulsive ADHD presentations.

### Study contributions

Despite these limitations, to our knowledge, this is the first study to evaluate mediation effects in the relationships between the EF, FFM personality traits, and ADHD in adolescents. Although some studies have shown relationships between the FFM traits, EF and ADHD, the links remain unclear. This may be due to individual differences in personality traits and measuring instruments, as well as the heterogeneity in both executive profiles and ADHD symptoms. Our findings support the hypothesis regarding EF deficits and the configuration of the FFM personality traits in ADHD, building on previous research in terms of understanding the multiple causal pathways associated with the heterogeneity of ADHD. As such, the results suggest that a method combining the use of personality questionnaires, EF measures and rating scales of ADHD symptoms is probably helpful to improve the individual characterization of functional personality attributes [99] in ADHD adolescents. More research is now need in this area. Future studies should seek to relate personality measures and cognitive tests, since these two types of measures are not often used within the same study, probably because they belong to two different research traditions with specific methodologies and assessment approaches.

## Supporting information

S1 Table Summary and descriptive statistics of cognitive executive functions (EF) measures (*N* = 118). (DOCX)
