# Supplementary material for: Executive functions, Personality traits and ADHD symptoms in adolescents: A mediation analysis
Source: PLoS One. 2020 May 6;15(5):e0232470. doi: 10.1371/journal.pone.0232470 (PMC7202790; doi:10.1371/journal.pone.0232470)
Supplement: S1 Table — (DOCX) [file pone.0232470.s001.docx]

| Summary and descriptive statistics of cognitive executive functions (EF) measures (*N* = 118). | | | | |
| --- | --- | --- | --- | --- |
| Cognitive EF | Performance-based tasks | Score type | ADHD  (75)  Mean (SD) | Controls  (43)  Mean (SD) |
| Visuospatial Working memory | Wechsler Nonverbal Scale of Ability (WNV): Spatial span (SSp) | SSp: Forward^#^ | 5.70 (.99) | 6.21 (.86) |
|  |  | SSp: Backward^#^ | 5.17 (.92) | 5.58 (.73) |
|  | Rey-Osterrieth Complex Figure Test ( ROCF) | Immediate recall accuracy^#^ | 15.46 (6.16) | 19 (6.46) |
| Flexibility | Wisconsin Card Sorting Test (WCST) | Perseverative errors ^*^ | 105.57 (16.94) | 104.63 (14.32) |
|  |  | Conceptual level responses ^*^ | 100.79 (14.68) | 104.14 (16.66) |
|  |  | Number of categories completed^#^ | 5.41 (1.12) | 5.53 (.98) |
|  | Trail Making Test (TMT) | Total time in seconds part B^#^ | 100 (34) | 77.40 (30.81) |
| Inhibition | Porteus Maze Test (PMT) | Qualitative *Q* score^#^ | 22.77 (11.48) | 14.05 (8.21) |
|  | d2 Test of Attention | Commission errors^#^ | 5.25 (9.69) | 1.53 (1.98) |
|  |  | Total test effectiveness^#^ | 323.88 (76.37) | 389.70 (68.49) |
| *Note.* ^#^*Rs* = raw score; ^*^ = T- score. | | | | |
